# Inferring Variation in Copy Number Using High Throughput Sequencing Data in R

**DOI:** 10.3389/fgene.2018.00123

**Published:** 2018-04-13

**Authors:** Brian J. Knaus, Niklaus J. Grünwald

**Affiliations:** Horticultural Crops Research Unit, United States Department of Agriculture-Agricultural Research Service, Corvallis, OR, United States

**Keywords:** bioinformatics, computational biology, copy number variation (CNV), high throughput sequencing (HTS), *Phytophthora*, ploidy, R package

## Abstract

Inference of copy number variation presents a technical challenge because variant callers typically require the copy number of a genome or genomic region to be known *a priori*. Here we present a method to infer copy number that uses variant call format (VCF) data as input and is implemented in the R package *vcfR*. This method is based on the relative frequency of each allele (in both genic and non-genic regions) sequenced at heterozygous positions throughout a genome. These heterozygous positions are summarized by using arbitrarily sized windows of heterozygous positions, binning the allele frequencies, and selecting the bin with the greatest abundance of positions. This provides a non-parametric summary of the frequency that alleles were sequenced at. The method is applicable to organisms that have reference genomes that consist of full chromosomes or sub-chromosomal contigs. In contrast to other software designed to detect copy number variation, our method does not rely on an assumption of base ploidy, but instead infers it. We validated these approaches with the model system of *Saccharomyces cerevisiae* and applied it to the oomycete *Phytophthora infestans*, both known to vary in copy number. This functionality has been incorporated into the current release of the R package *vcfR* to provide modular and flexible methods to investigate copy number variation in genomic projects.

## Introduction

Investigations into the variation in the number of copies of genes, chromosomes, or genomes are well-established research topics, yet they continue to present technical challenges to molecular genetic analysis. Many examples provide evidence of how copy number affects the phenotype. For example, schizophrenia in humans is thought to be caused by variation in copy number of certain genes (Sekar et al., 2016). Presence of an additional chromosome (aneuploidy) results in Down syndrome in humans (Hassold and Hunt, 2001). Existence of an extra copy of all chromosomes (triploidy) is used in agriculture to produce sterile organisms such as seedless watermelons (Varoquaux et al., 2000) or sterile salmon (Johnstone, 1992; Cotter et al., 2000). Whole genome duplication (polyploidy) results in every chromosome being duplicated, a phenomenon observed throughout plants, animals, and fungi (Todd et al., 2017; Van de Peer et al., 2017). Although this phenomenon is well established, it presents a challenge to high throughput sequencing projects in that most popular genomic variant callers, such as the GATK’s (DePristo et al., 2011) or FreeBayes (Garrison and Marth, 2012), require the *a priori* specification of how many alleles to call. While the inference of copy number may be an important precursor to point mutation discovery, many authors argue that copy number variation may be more abundant throughout a genome than point mutations (Katju and Bergthorsson, 2013) making it an important facet in the investigation of genomic architectures.

**FIGURE 1:**
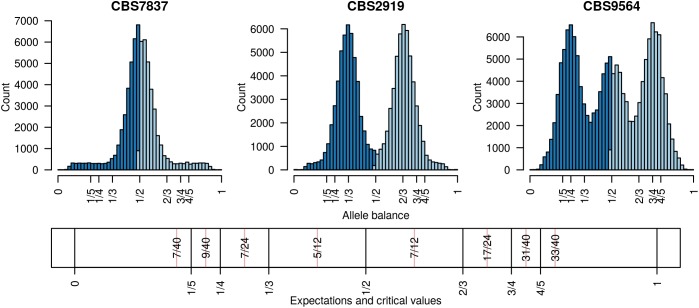
Allele balance (e.g., the distribution of the frequency at which the most abundant allele and the second most abundant allele were sequenced) at heterozygous positions in three *Saccharomyces cerevisiae* genomes. For each heterozygous genotype the frequency at which the most abundant allele was sequenced at (light blue) and the frequency at which the second most abundant allele was sequenced at (dark blue) were recorded. This information was then summarized with a histogram. Expectations for allele balance are 1/2 for diploids, 1/3 and 2/3 for triploids, and 1/4, 1/2, and 3/4 for tetraploids. This approach provides a dominant copy number for each genome but no information about variation within each genome. Expectations and critical values for binning allele balance information are presented below the histograms.

Existing software for determining the number of copies at a locus from high throughput sequencing data can be broadly classified into two categories: copy number variation detection and whole genome ploidy inference. The important difference among these categories is the form of data they use. Copy number variation detection software uses per position sequence depth (Yoon et al., 2009; Abyzov et al., 2011; Klambauer et al., 2012; Li et al., 2012) while whole genome ploidy inference software uses the relative frequency of the two most abundant alleles sequenced at a locus (Zohren et al., 2016; Gompert and Mock, 2017; Weiß et al., 2018). Copy number variation detection methods group the per position sequence depth into windows and attempt to sort these into base-ploid (typical depth) windows or windows that deviate from base-ploid. They generally require the investigator to specify *a priori* what copy level the base-ploid state is. If the research question is to determine how many copies occur at the base-ploid state, these methods will not be appropriate. Whole genome ploidy inference methods use the frequency that the two most abundant alleles were sequenced at for heterozygous positions, or allele balance, and summarize this information throughout the genome. (Here we use the term ‘allele balance’ where other authors have used ‘allele frequency’ to distinguish the measure from the use of ‘allele frequency’ in population genetics.) For example, for heterozygous alleles we would expect to observe an approximate frequency of one half for diploids, ratios of thirds for triploids, and ratios of quarters for tetraploids (**Figure 1**). Whole genome ploidy inference uses all of the genomic information to infer a single copy number for the entire genome. A third hybrid method uses allele balance (referred to as allelic ratio) and heterozygosity to assign copy number to populations of data (McKinney et al., 2017). However, if the research question is to explore copy number variation within a population this method will not be relevant. Therefore, there are at least two distinct approaches to determine the number of copies present in genomes, and more currently being proposed, each with different strengths and limitations.

Our research presented us with the need to determine if copy number varied throughout genomes, where we did not have prior knowledge of what the actual base-ploidy might be. We therefore combined the windowing functionality from copy number variation detection methods with the allele balance concept from whole genome ploidy inference methods. We use a non-parametric approach to infer copy number given that empirical explorations of available data indicated that common distributions, particularly at low sequence depth, do not fit well. Our method is implemented in a new update to the package *vcfR* in the R software environment (R Core Team, 2018). R is an established and growing language facilitating the analysis of population genetic and genomic data (Paradis et al., 2017a,b). We demonstrate the utility of this method using genomes from the model fungus *Saccharomyces cerevisiae* and our ongoing work with the oomycete plant pathogen *Phytophthora infestans*. Both of these organisms show variation in ploidy across individuals as well as within regions within a genome.

## Materials and Methods

### Methodology

We developed new functionality added to the current release of the *vcfR* package that can be used to infer copy number or ploidy in R. We initially developed *vcfR* for VCF data import/export, quality control, visualization and general manipulation (Knaus and Grünwald, 2017). *vcfR* now includes a range of new functions useful for binning variants into windows, summarizing the frequency that alleles were sequenced at, and assigning a closest expected copy number value to these windows (**Table 1**).

**Table 1:** Functions available to analyze copy number variation and mixed copy number data in the current release of *vcfR*.

Function	Description
extract.gt()	Isolate data from the delimited VCF genotype fields.
freq_peak()	Windowize and identify peaks of density.
is_het()	Identify heterozygous variants.
masplit()	Isolate values from a matrix of delimited data.
peak_to_ploid()	Convert peaks of density to an expected copy number.
freq_peak_plot()	Visualize results from freq_peak().
rePOS()	Convert chromosomal positions to genomic (non-overlapping) positions.
genetic_diff()	Calculate genetic differentiation (*G*_ST_).


Data from high throughput sequencing (HTS) projects on populations typically results in calling variants that might include single nucleotide polymorphisms (SNPs), indels, and inversions. Output from popular variant callers is presented in files that adhere to the variant call format (VCF) specification (Danecek et al., 2011). This specification provides the option to include counts for how many times each allele was sequenced for each genotype. For example, in the GATK’s HaplotypeCaller (McKenna et al., 2010) output includes allele depth (AD) as a comma delimited string of counts. This VCF data can be imported into R using our function read.vcfR(). Once any desired quality control steps have been performed on the data (Knaus and Grünwald, 2017), such as omitting variants of unusual sequence coverage, this allele depth data can be extracted using the *vcfR* function extract.gt(). We then use the function is_het() to set homozygous positions in the allele depth matrices as missing data (NA) so we can focus our analysis on the heterozygous positions. The allele depth is reported as a comma delimited string, the individual elements of which can be isolated with the function masplit(). Dividing the count for each allele by the sum of the counts for the two most abundant alleles, results in the frequency at which each allele was sequenced, or allele balance. This data can now be plotted as histograms for visualization.

Determining copy number for sub-genomic regions requires the genome to be divided into sub-genomic windows and, because this typically results in many windows per sample, it requires a numeric method of summarizing this data. This goal is accomplished with the function freq_peak(). This function takes as input a matrix of allele balance data, as described above, a vector of chromosomal positions for each variant, a window size, and a bin width for summarizing the allele balance values. The vector of chromosomal positions is used to assign variants to windows. The window size specifies how large the genomic windows should be. This will in part be based on the frequency of heterozygous positions observed in the target sample as well as a balance between the conflicting desires for small windows that provide fine scale resolution and large windows that provide a large number of variants (i.e., support) for a determination. Within each window the allele balance values are summarized by bins from 0 to 1 and of the width specified by the bin width parameter. The bin with the greatest number of variants is selected as the peak location. Here, again, a balance must be found between resolution (small bins) and support (large bins). Default values are provided based on what we have determined to work in our study systems, but we highly encourage adjusting the parameters based on the specifics of each project. These parameters are expected to be context specific to each study system. This function returns three matrices, one containing the window coordinates, one containing the peak locations and one containing the count of variants that resulted for each window. The matrix of variant counts per window can be used to help determine optimal window size and to censor windows that resulted in a low number of variants. The peaks can then be assigned to their nearest expected value (1/5, 1/4, 1/3, 1/2, 2/3, 3/4, 4/5) using the function peak_to_ploid(). This is accomplished by using critical values that are half way between each expected value (**Figure 1**). Once a copy number has been assigned its confidence is measured by creating a distance from expectation. The distance from expectation is the observed value subtracted by the expectation it was assigned to which is then divided by the critical value on the side of the expectation where the observed value was (**Figure 1**). Dividing the critical value scales the difference from expectation from zero (exactly at our expectation) to one (half way between expectations). This can also be used to remove border cases where observed value is intermediate to the expected values and we therefore have low confidence in the determination. The results from the function freq_peak() can be visualized using freq_peak_plot(). This last function was inspired in part by BAF plots (Laurie et al., 2010).

Theoretical population genetics is based largely on haploid and diploid organisms. Investigations into populations that consist of higher ploidy individuals, or populations with a mixture of copy numbers, present a methodological challenge in that few applications are available to analyze them. We have extended Nei’s *G*_ST_ (Nei, 1973, 1987) and Hedrick’s *G*’_ST_ (Hedrick, 2005) to address this challenge. These measures of population subdivision are based on ratios of heterozygosity. Because heterozygosity is based on the number and type of alleles found in a population it provides a convenient way to analyze populations of mixed copy number. Our implementation is inspired by the implementation in *adegenet* (Jombart, 2008) which weights the heterozygosities by their sample size. This is an attempt to correct for unbalanced sample sizes, situations where a different number of individuals were sampled from different populations. We instead weight the heterozygosities by the observed number of alleles in each population to correct for both unbalanced samples as well as instances where individuals may vary in copy number as well. An unbalanced design occurs when different amounts of data are collected for different populations. For example, one sample may have consisted of 20 individuals while another may have only consisted of 10. This imbalance may have occurred due to logistical reasons or technical issues in sample preparation. When copy number is unknown, the investigator may sample the same number of individuals in the populations, but if one population turns out to have four copies where the other has only two, the population with four copies will have twice as much information as the other. Weighting each population by the number of alleles observed is an attempt to mitigate these issues. The function genetic_diff() uses a *vcfR* object and a factor that indicates population membership (VCF data typically does not include population information) and returns a table including heterozygosities, Nei’s *G*_ST_, and Hedrick’s *G*’_ST_.

### Example Data

To demonstrate our method, we tested it on three data sets. The first data set consisted of three samples of *Saccharomyces cerevisiae* (CBS7837, CBS2919, and CBS9564) from Zhu et al. (2016) that were reported as diploid, triploid and tetraploid by Weiß et al. (2018). We also included an additional sample (YJM1098) that was reported by Zhu et al. (2016) as being predominantly diploid but demonstrating aneuploidy for chromosome XII. These samples represent an organismal system where the genome is of relatively small size (12 Mbp), high quality (in its 64th revision; Engel et al., 2014) and where the samples were sequenced with a goal of attaining 80X sequence depth with Illumina GAII reads.

A second data set consisted of two samples of the plant pathogen *Phytophthora infestans* (99189 and 88069) that were reported by Weiß et al. (2018) as being diploid and triploid. The *P. infestans* system represents a more modestly sized genome (240 Mbp) that remains in its first draft (Haas et al., 2009), but where the samples were sequenced with the intent of attaining 100X sequence depth for each haplotype using Illumina HiSeq 3000 sequencing (Weiß et al., 2018).

The third dataset included 17 samples of *P. infestans* and one sample of *P. mirabilis* collected from the literature, subset to Supercontig_1.50, and made available as an R package (Knaus and Grünwald, 2017). This represents a set of samples that were of more typical sequence depth for genomics projects than we might expect from investigations that were specifically interested in copy number.

For the first two datasets, the data were downloaded from the NCBI sequence read archive and FASTQ data were extracted using the sratoolkit. These reads were mapped to the yeast genome (S288C) or the *P. infestans* genome (T30-4) using bwa 0.7.10-r789 mem (Li, 2013). The resulting SAM file had mate pair information updated, was sorted and converted to BAM format using samtools 1.3.1 (Li et al., 2009). Duplicates were marked using picard-tools-2.5.0 and the files were indexed using samtools. For each sample, a g.VCF file was created from its BAM file using the GATK’s (3.5-0-g36282e4) HaplotypeCaller (McKenna et al., 2010). Read processing for the pinfsc50 was described previously (Knaus and Grünwald, 2017). Briefly, the reads were mapped using bwa mem and variants were called using the GATK’s HaplotypeCaller resulting in VCF data. The g.VCF and VCF data were processed in *vcfR* (Knaus and Grünwald, 2017) using the methods described above using the functions freq_peak(), peak_to_ploid(), and freq_peak_plot(). For the *S. cerevisiae* samples, a window size 40 kbp was used while a window size of 200 kbp was used for the *P. infestans* samples.

### Performance

We assessed performance of our method over a range of genome sizes. Data used for the benchmarking were subset from the 99189 *P. infestans* sample including the entire data set (240 Mbp genome) and subsets of this dataset to represent genomes of 100, 10, and 1 Mbp. Each data set was processed 20 times and this processing was implemented using an R markdown script. The use of R markdown, as opposed to a pure R script, likely incurred a performance cost as our timing included the compilation of the R markdown to a web page. We advocate that using tools like R markdown should be considered a best practice and hope that this will characterize typical use. Benchmarking was performed on an Intel© Core^TM^ i7-4790 CPU at 3.60 GHz with 32 GB of RAM running Ubuntu 16.04 LTS. Results were visualized in R and a linear regression was performed using the R function stats::lm().

## Results

### Implementation

A new update for the R package *vcfR* was recently released including several new functions (**Table 1**). The function freq_peak() returns the peaks called for each window as well as diagnostic information. The data in VCF files only includes information for the variable positions. This means that all positions in a window will not be present in VCF data. A lookup table is created and returned that includes the genomic coordinates for each window, the row number of the first and last rows of VCF data that were analyzed, and the genomic position of the first and last variant in each window. This information is intended to coordinate comparisons among data extracted from VCF files and genomic windows. A matrix of variant counts per sample and window is also provided. Because heterozygosity may not be known and some windows may have mapping issues (e.g., high variant counts) or regions of loss of heterozygosity or a high number of missing or ambiguous nucleotides in the reference (low variant counts), this information can be used to help determine optimal window size for a particular organism. Furthermore, this approach can help identify anomalous regions in the genome that may require further scrutiny. Lastly, a matrix of frequencies of allele balance is generated.

Results of the above process can be visualized and post-processed to obtain copy number calls and quality assessment. The function freq_peak_plot() can be used to visualize the combined VCF derived data and the results of the windowing and peak calling operations. Because the result is a simple data structure (a list of matrices) the universe of R packages that can be used with matrix data are also available to explore the data. The data can also be post processed with the function peak_to_ploid() that converts the allele balance frequency data to an integer copy number as well as distances from expectation:

$$\text{Distance from expectation=}\frac{\text{observed allele balance − expected value}}{\text{critical value}}$$

The distance from expectation is the observed allele balance frequency subtracted by the frequency expected based on the final determination. This value is then divided by its bin width (**Figure 1**) in order to scale it from zero to one where zero represents an allele balance that is exactly on our expectation (e.g., 1/4, 1/3, 1/2, etc.) and one is half way between two expectations. This value can then be used as a measure of confidence in our copy number determination and to omit border cases (instances where the observed allele balance is close to one).

### *Saccharomyces cerevisiae* Dataset

**FIGURE 2:**
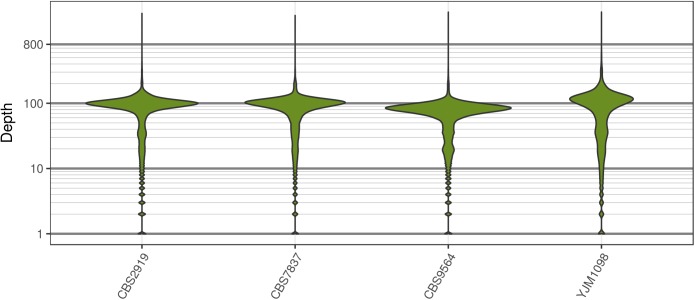
The distribution of sequence depth at variable positions in *Saccharomyces cerevisiae*. While each genome was sequenced at close to 100X, each genome also had long tails for variants that were sequenced at very high and low coverage. These tails are typically observed for high throughput sequencing data.

**FIGURE 3:**
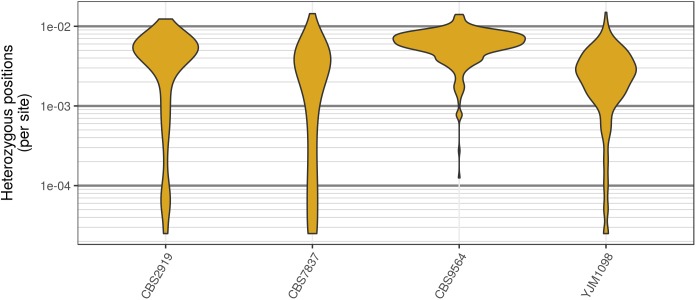
Genomic distribution of heterozygous positions in *Saccharomyces cerevisiae* genomes. Each genome was divided into 40 kbp windows, the number of variants was counted within each window, and this count was divided by the window size. While most windows had a typical number of heterozygous positions (2–8 per kbp) there were a substantial number of windows that contained very few heterozygous positions. Note that these are raw variants from the VCF file produced by the variant caller (in our case, GATK HaplotypeCaller). Because most variant callers take an aggressive perspective on variant calling, the values presented are likely an over-estimate of heterozygosity.

**FIGURE 4:**
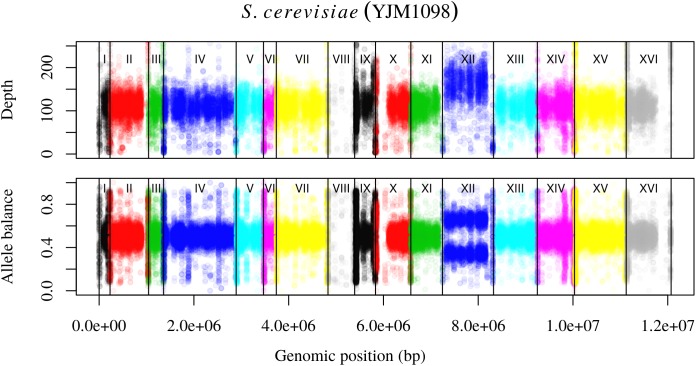
Reproduction of Figure 7 from Zhu et al. (2016). The upper panel demonstrates the concept of base ploidy where most of the genome is of one ploidy however, we do not know how many copies this base ploidy consists of. The lower panel demonstrates how allele balance is predominantly what we would expect for a diploid, allowing us to assign a copy number to the base ploid. Chromosome XII demonstrates a change in copy number that is evident as a change in base ploidy and allele balance.

**FIGURE 5:**
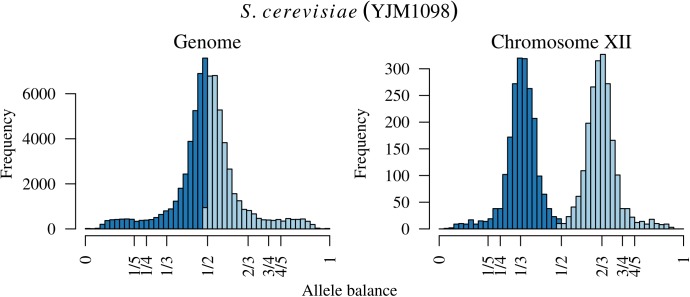
The distribution of allele balance values for an entire sample of *Saccharomyces cerevisiae* and the distribution for just chromosome XII. Note the *y*-axis for each plot. The distribution on the right is contained within the distribution of the entire sample on the left so that this variation in copy number is hidden in plain sight.

**FIGURE 6:**
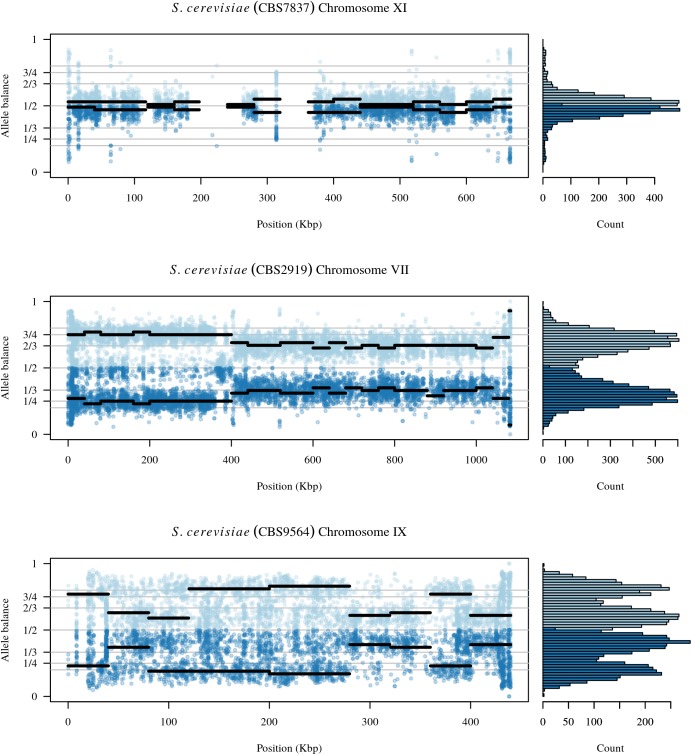
The chromosomal distribution of heterozygous positions and their allele balance. Each plot represents one chromosome. At each variable position along the chromosome there is a pair of dots: a light blue dot above 1/2 and a darker blue dot below 1/2. These dots are the allele balance for each variant. Horizontal lines represent windows where the width is the user specified window size and the elevation is the summarized allele balance for the window. The marginal histogram summarizes the entire chromosome. The top plot is chromosome XI from sample CBS7837 and represents a diploid example. Regions at 230 and 350 kbp are regions that exhibit low levels of heterozygosity and the lack of a horizontal line indicates that these regions were omitted from the results. The middle panel is from chromosome VII of sample CBS2919. This chromosome appears to consist of four copies from its origin to around 400 kbp where it changes to three copies. The bottom panel is chromosome IX from sample CBS9564. This chromosome appears to consist of regions that have three copies as well as regions with five copies.

Analysis of the *Saccharomyces cerevisiae* dataset validated previous reports and revealed new features. The *S. cerevisiae* samples were sequenced at about 100X at variable positions (**Figure 2**) making it a high coverage dataset. The samples were determined to consist of individuals that were predominantly diploid (CBS7837), triploid (CBS2919), and tetraploid (CBS9564), confirming previous reports (**Figure 1**; Weiß et al., 2018). The samples had a heterozygosity of around 0.003–0.008 heterozygous positions per site (**Figure 3**). Because the variant caller (the GATK’s HaplotypeCaller) tends to aggressively call variants, this estimate may include false positives and therefore may be an overestimate of the true biological value. We have previously discussed strategies we feel may improve the quality of called variants to attain a production data set (Knaus and Grünwald, 2017). Current functionality in *vcfR* allowed for convenient reproduction of figures previously reported (**Figure 4**; Zhu et al., 2016) that indicated intragenomic variation in copy number. This copy number variation was demonstrated to be minor relative to the entire genome (**Figure 5**), indicating that while sample YJM1098 may be predominantly diploid, it still contains variation that would not be apparent from whole genome summaries. The use of the *vcfR* functions freq_peak() and peak_to_ploid() provided a sliding window analysis that revealed intragenomic variation in copy number. **Figure 6** demonstrated the results of the function freq_peak_plot() that revealed a sample that appeared diploid, but contains regions of low heterozygosity such that inferences cannot be made (CBS7837 chromosome XI at around 200 kbp and around 350 kbp). The sample CBS2919 appeared predominantly triploid, consistent with previous findings (Weiß et al., 2018), but also included a region on chromosome VII from its origin to around 400 kbp that appeared to have four copies. The sample CBS9564 was reported by Weiß et al. (2018) to be tetraploid, which is in agreement with our results, but also appeared to have regions on chromosome IX that had three or five copies. These findings confirm previous reports and also reveal that new information can be found by investigating specific regions within each genome.

### *Phytophthora infestans* Dataset

**FIGURE 7:**
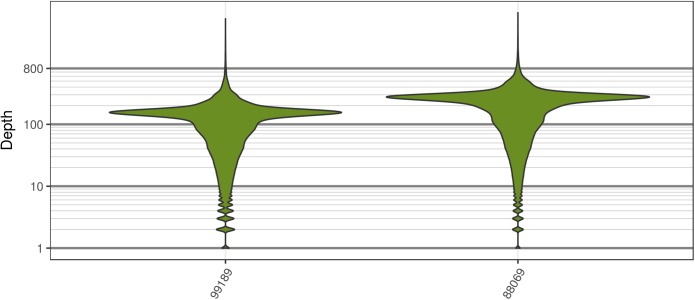
The distribution of sequence depths at variable positions for *P. infestans* samples produced by Weiß et al. (2018). These plots are similar to the *S. cerevisiae* plots in that most of the genome appears to have been sequenced at a base ploidy level, but long tails indicate that regions above and below this level exist.

**FIGURE 8:**
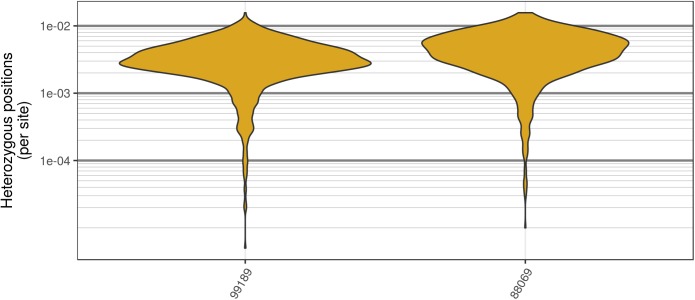
Genomic distribution of heterozygosity among genomic windows for the two *P. infestans* samples sequenced by Weiß et al. (2018). Each genome was divided into 200 kbp windows, the number of heterozygous positions were counted, and this count was divided by the window size. The *P. infestans* genome consists of 4,921 supercontigs, many of which were below the size of these windows. In order to mitigate this, only supercontigs that resulted in at least two windows are summarized here. Note that these are raw variants from the VCF file produced by the variant caller (in our case GATK HaplotypeCaller). Because most variant callers take an aggressive perspective on variant calling, the values presented are likely an over-estimate of heterozygosity.

**FIGURE 9:**
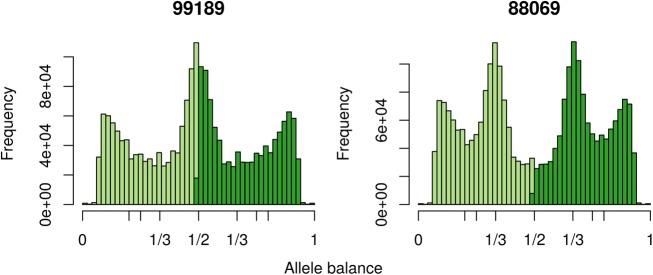
The distribution of allele balance frequencies for samples sequenced by Weiß et al. (2018). This graphically validates the ploidy levels reported by Weiß et al. (2018).

**FIGURE 10:**
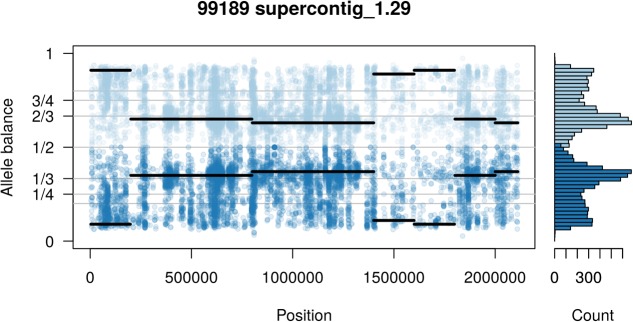
Supercontig_1.29 of *P. infestans* isolate 99189 appears predominantly triploid in contrast to the rest of its genome that appeared to be diploid (compare with **Figure 9**). Values of 0 (no read support for the allele) and 1 (all reads support one allele) are expected to be homozygous calls. Because this is an analysis of heterozygous positions these have been omitted from this plot.

The two *P. infestans* samples were sequenced at almost 200X (99189) and 300X (88069) or approximately 100X per expected chromosome (**Figure 7**; Weiß et al., 2018). The genomes had heterozygosities of around 0.003–0.006 heterozygous positions per site (**Figure 8**). Because the variant caller tends to aggressively call variants, this estimate may include false positives and therefore may be an overestimate of the true biological value. Examination of the genomic distribution of allele balance values confirmed the report of Weiß et al. (2018) that isolate 99189 was predominantly diploid while 88069 was predominantly triploid (**Figure 9**). However, through windowing across the supercontig, we were able to observe that while isolate 99189 does appear to be predominantly diploid, a large portion of its supercontig_1.29 appears to have three copies (**Figure 10**) demonstrating previously uncharacterized intragenomic variation in copy number.

### Pinfsc50 Dataset

**FIGURE 11:**
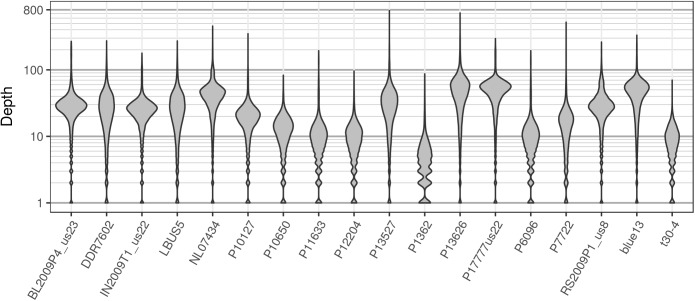
The distribution of sequence depths at variable positions for *P. infestans* samples from the pinfsc50 dataset with variants called for supercontig 50.

**FIGURE 12:**
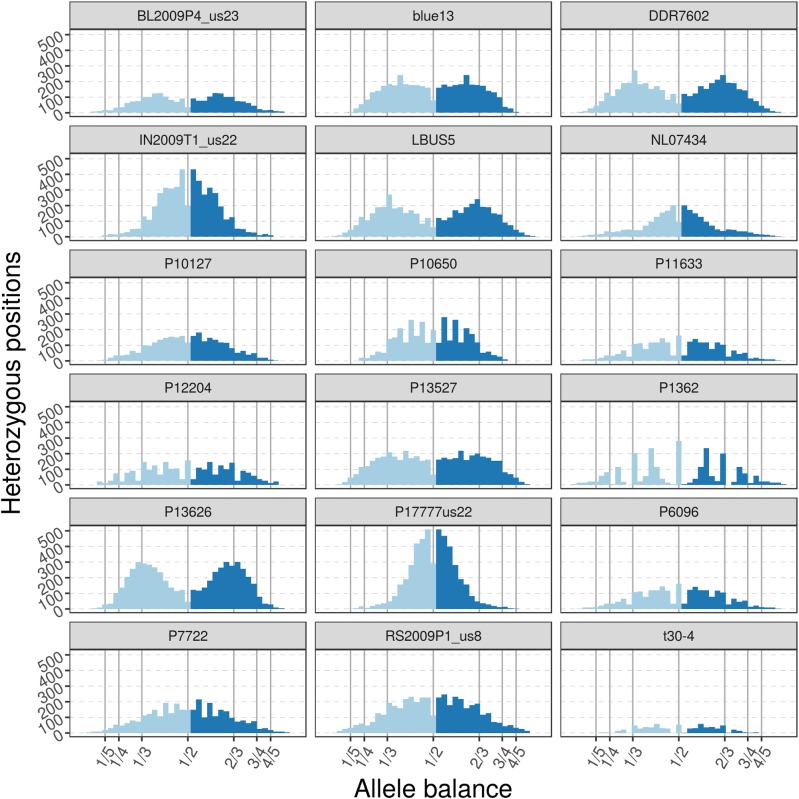
The distribution of allele balance values for variants from supercontig_1.50 of *P. infestans*. These samples are of a more typical read depth than the other samples presented here. Note that some samples may not have a copy number that is easily determined. This illustrates the importance of providing numerical summaries as well as visualizations for the data that demonstrate edge cases as well as methods to address poor quality (e.g., removal of data based on read depth thresholds).

The pinfsc50 dataset provides an opportunity to evaluate data with more moderate and more typical lower read depths. This data represents samples for a population of *P. infestans* at supercontig 50 that were sequenced between ca. 10X to 70X coverage (**Figure 11**). The distribution of allele balance values for these samples (**Figure 12**) demonstrated a range of copy numbers from diploid (e.g., strain P17777us22) to triploid (strain P13626). However, several samples (e.g., strains P1362 or t30-4) appeared to be ambiguous as to their copy number. This demonstrates that not all samples that have been sequenced from typical sequencing projects may be of suitable quality for copy number determination.

### Population Differentiation

The function genetic_diff() calculates genetic differentiation for mixed copy number populations (**Table 2**). It retains the chromosome and position information from the VCF data to maintain the coordinate system. Heterozygosities as well as the number of alleles observed in each population are returned. If the number of alleles in data are unknown, this latter information may be used to summarize this information. For larger data sets, quantiles can be calculated to identify loci of unusual allele counts. The function reports *G*_ST_, maximum heterozygosity, maximum *G*_ST_ and uses these to calculate *G′_ST_*. The returned data structure is a simple data.frame which should easily facilitate further analysis and presentation of this information with the universe of R functionality.

**Table 2:** Genetic differentiation as reported by the function genetic_diff().

CHROM	POS	Hs_a	Hs_b	Ht	n_a	n_b	Gst	Htmax	Gstmax	Gprimest
Supercontig_1.50	2	0.42	0.42	0.4650	20	20	0.096	0.710	0.408	0.237
Supercontig_1.50	246	0.42	0.42	0.4632	20	30	0.093	0.698	0.399	0.234
Supercontig_1.50	549	0.42	0.42	0.4600	20	40	0.0870	0.678	0.380	0.229


*The chromosome (CHROM) and position (POS) are retained from the VCF data. Heterozygosities for each population (a and b) and total heterozygosity are reported. The number of alleles (n_a, n_b)_observed in each population are reported. Lastly, *G_*ST*_*, maximum heterozygosity (Htmax), maximum *G_*ST*_* (Gstmax) and *G′_*ST*_* (Gprimest) are calculated.*

### Performance

**FIGURE 13:**
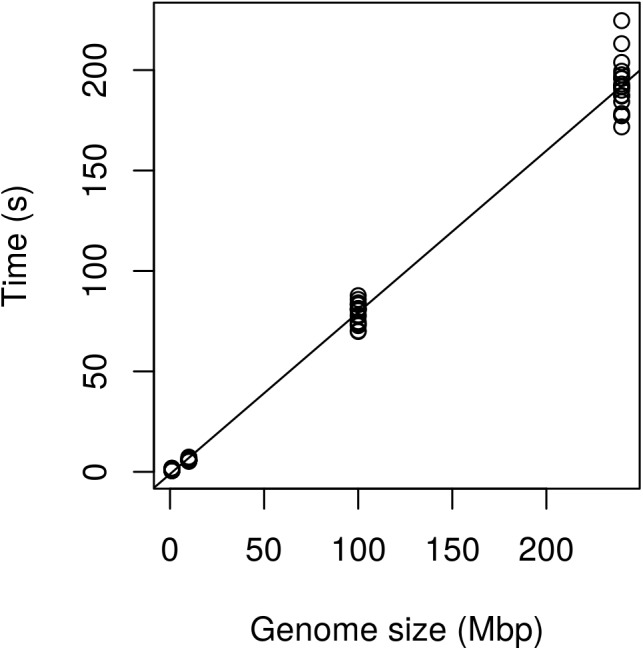
Performance of the method expressed as execution time (seconds) as a function of genome size (Mbp). The Genome of *P. infestans* 99189 was used and subsampled at 100, 10, and 1 Mbp. Performance appears to scale linearly with the 240 Mbp genome being processed in just over 3 min.

Regression analysis revealed that execution time scaled linearly with genome size (**Figure 13**). There was a highly significant relationship between execution time and genome size (**Table 3**) indicating that our benchmarking may be a good predictor of how the method will perform with other genomes.

**Table 3:** Coefficients resulting from the linear regression of execution time (seconds) as a function of genome size (Mbp).

Coefficient	Estimate	Standard error	*t*-value	*P*-value
Intercept	-1.085	1.010	-1.075	0.286
Slope	0.805	0.008	103.663	<2e-16


*The intercept was not significantly different from zero while the slope was highly significantly different from zero.*

## Availability

Version 1.7.0 of the package *vcfR* had been released at the time of submission of this manuscript and contains all of the novel features described here. This version is available on CRAN (https://CRAN.R-project.org/package=vcfR) and at the Grünwald lab’s GitHub site (https://github.com/grunwaldlab/vcfR). More information and example code can be found at: https://knausb.github.io/vcfR_documentation/. Data and scripts used to produce figures in this manuscript are available at the project’s Open Science Framework site (Knaus and Grünwald, 2018).

## Requirements

• R version 3.0.1 or greater and *vcfR* 1.7.0.

## Installation

At the R console, *vcfR* can be installed from CRAN as follows:

install.packages(‘vcfR’)

library(‘vcfR’)

## Discussion

Numerous studies have used high throughput sequencing to study genetic diversity in populations based on genotypes, or single nucleotide polymorphisms, inferred by variant callers. To our knowledge there is currently no variant caller that can infer the number of alleles to call. Instead, the investigator must specify the number of alleles to call *a priori*. Here we present novel methodologies to infer genomic and subgenomic copy number using HTS data as well as to visualize these data in the R environment.

Our method builds on existing methods by using a sliding window approach to infer copy number based on the frequency that the most abundant and second most abundant alleles were sequenced at. While we designed this method to work with VCF data (Danecek et al., 2011) using the R package *vcfR* (Knaus and Grünwald, 2017), we feel an important role of our method is to help make this data available to the existing universe of R packages. VCF data only includes information on variable positions within the genome. We therefore produce a lookup table to identify which genomic windows variants belong to. Other functions convert the VCF data into numeric matrices. In theory, this information could be used to implement other functionality, such as applying mixture models (Leisch and Gruen, 2012; Fraley et al., 2012) to the data. It also means that other visualization tools available to the R environment can be used beyond those provided here. Because characterization of copy number may be challenging in certain regions of the genome, e.g., regions rich in transposable elements or problematic assemblies, we provided the count of heterozygous positions for each window as well as the distance from expectation. These metrics provide tools to help judge whether certain regions may have well predicted copy numbers or which regions may require further investigation.

The existing methods most similar to ours include those of Zohren et al. (2016), Gompert and Mock (2017), and Weiß et al. (2018) because they are all based on the frequency that alleles were sequenced at. Zohren and colleagues used allele balance (which they referred to as allelic ratio) and fit beta-binomial distributions to model diploid individuals and beta-binomial mixture models (the fitting of multiple distributions to a population of data) to model triploid and tetraploid individuals. Likelihoods for each ploidy model were compared using AIC (Akaike, 1974), resulting in a single ploidy call for each sample. R code to implement their method is available at Dryad. Gompert and Mock model the ratio of the abundance of the non-reference allele (from biallelic SNPs) to the total number of reads sequenced at each variant using binomial distributions in a Bayesian framework resulting in a single ploidy call for each sample. Their method is implemented in R using *rjags* (Plummer, 2016) and is available on CRAN as the package *gbs2ploidy*. The method of Weiß and colleagues is similar to that of Zohren and colleagues in that it employs mixture models; however, it differs in that it uses Gaussian components. It also differs in that it is written in C and designed to work on the BAM files as opposed to heterozygous positions determined by a variant caller. Because it is implemented in a compiled language it is very fast relative to the R implementations. It is also unique in that it employs a uniform noise component. The sample CBS7837 in **Figure 1** has a well-defined peak, yet the base of the peak varies almost from zero to one indicating a substantial amount of data that deviates from any of our expectations. Similarly, the sample CBS2919 in **Figure 1** has two well defined peaks but the data does not go to zero between these peaks. This phenomenon can be seen in Zohren and colleagues’ **Figure 2** and Yoshida et al. (2013) **Figure 8** and is part of our justification for the use of a non-parametric method. Weiß and colleagues fit this uniform component in an attempt to capture the noise in the data leaving the putatively cleaner data for their Gaussian mixture model. Their software is available on GitHub in the repository named nQuire.

The method presented has been designed to work with VCF data (Danecek et al., 2011) that contains the number of times each allele was sequenced for each variant. In theory, any method that produces a valid VCF file, or the counts of times the most abundant and second most abundant allele were sequenced in a format that can be read into R, can be analyzed. While the examples presented here are based on whole genome sequencing our method should be applicable to data generated with reduced representation libraries. For example, we’ve also used the method with genotyping-by-sequencing data (Elshire et al., 2011) processed with TASSEL (Bradbury et al., 2007). However, there are some practical matters to consider. This is an analysis of heterozygous positions. Homozygous positions will appear similar regardless of copy number and are uninformative. Organisms that are inbred or have a mode of reproduction that includes selfing may have a low density of heterozygous positions making inferences using our method challenging. The use of reduced representation libraries may also contribute to a lower number of observed heterozygous positions requiring use of larger windows ultimately resulting in a lower resolution to the inference of copy number variation.

There is currently a diversity of methods available for the analysis of high-throughput sequencing that demonstrates a diversity of performance. This diversity in performance exists in *de novo* assembly software (Earl et al., 2011; Bradnam et al., 2013), variant callers (Pabinger et al., 2014), copy number variation callers (Duan et al., 2013; Pabinger et al., 2014), and metagenomic pipelines (Edgar, 2017). This diversity is likely due to the nascent nature of the data and methods used to analyze it. We hope our method will contribute to the analysis of CNV, but also hope it will stimulate the development of new tools or the integration of these existing methods into new tools to explore copy number variation. Perhaps future improvements can be found by integrating sequence coverage and allele balance data as some authors have already done graphically (Zhu et al., 2016).

## Author Contributions

BK conceived the project, wrote code, wrote the documentation, and wrote the manuscript. NG conceived the project, coordinated the collaborative effort, discussed interpretation, wrote the manuscript, and obtained funding.

## Conflict of Interest Statement

The authors declare that the research was conducted in the absence of any commercial or financial relationships that could be construed as a potential conflict of interest.
